# A Case of Retrorectal Melanoma Presenting With Bleeding Per Rectum

**DOI:** 10.7759/cureus.83291

**Published:** 2025-05-01

**Authors:** Saurabh Dubey, Memoona Khan, Nasir Gondal

**Affiliations:** 1 Internal Medicine, Flushing Hospital Medical Center, New York City, USA; 2 Hematology-Oncology, Flushing Hospital Medical Center, New York City, USA

**Keywords:** bleeding per rectum, lower gi bleed, malignant melanoma, malignant melanoma initial presentation, mechanical large bowel obstruction, pelvic mass, pelvic melanoma, rectal malignant melanoma, retrorectal mass, stage iv melanoma

## Abstract

Rectal bleeding is a common presenting complaint in the emergency department (ED), most frequently due to benign conditions like hemorrhoids, anal fissures, and diverticulitis. Malignancies are a less common cause, with colorectal adenocarcinomas being more typical. Melanomas, malignant tumors of melanocytes, primarily occur on the skin but can arise in less visible sites, including mucosal surfaces and rarely as pelvic masses. We report a case of a 56-year-old female who presented to the ED with rectal bleeding. Initial examination did not reveal a mass, and a prior history of hemorrhoids led to an initial discharge without further workup. However, subsequent presentation with abdominal pain prompted further investigation, revealing significant leukocytosis and anemia. Imaging studies, including chest radiography and CT of the chest, abdomen, and pelvis, demonstrated multiple metastatic lesions in the lungs and liver, as well as a large retrorectal mass. Biopsies of a liver lesion and a rectal mass confirmed the diagnosis of metastatic melanoma.

The patient was treated with immunotherapy but experienced clinical deterioration and ultimately elected for comfort measures, passing away approximately three months after the initial presentation, likely due to bowel obstruction secondary to the retrorectal melanoma. This report highlights the rare presentation of a stage IV retrorectal melanoma with initial rectal bleeding, emphasizing the importance of considering unusual malignancies in the differential diagnosis of this common symptom, particularly when initial assessments are non-diagnostic.

## Introduction

Melanomas are malignant lesions arising from melanocytes, which are distinctive in their synthesis of melanin pigments [1]. Melanoblasts, precursor cells to melanocytes, migrate during fetal development throughout the dermis to the skin, the eye, the inner ear, and the leptomeninges [2]. A group of melanocytes will often form a nevus, a benign skin lesion, in many people. Melanomas of the skin are relatively easier to diagnose due to characteristic features distinguishing them from nevi, such as irregular borders, asymmetry, non-uniform color, and local symptoms like itching, which are easily visible and can be diagnosed with a simple physical exam. However, diagnosing melanoma arising from less visible areas, such as the pelvis, is more challenging. These patients often present with nonspecific symptoms and at a later stage of the disease. Symptoms vary based on the location of the lesions, with melanomas that form pelvic masses presenting with symptoms like abdominal pain, bloating, and weight loss [3,4,5]. The most common sites of metastasis are skin and subcutaneous tissue, followed by the lungs, liver, bones, and brain. The five-year survival rate for patients with metastatic melanoma diagnosed between 2012 and 2018 is around 30% [6]. We discuss the case of a patient who presented with a retrorectal melanoma leading to rectal bleeding, which prompted the initial investigation and workup.

## Case presentation

A 56-year-old female presented to the emergency department (ED) with rectal bleeding that had started one day prior. Physical examination revealed no significant findings, and a digital rectal exam at the time showed no palpable masses. Her past medical history included hemorrhoids, diagnosed via colonoscopy one year prior. Given this clinical history and presentation, she was initially discharged from the ED with recommendations for outpatient follow-up for presumed hemorrhoidal bleeding. No labs were drawn at this initial ED visit. The next day, she presented to a different ED for epigastric burning pain that began a day later. A summary of her Bloodwork is presented in Table 1.

**Table 1:** Results of initial bloodwork during subsequent ED visit ED: emergency department; MCV: mean corpuscular volume

Lab test	Result	Reference range
White blood cell count	41.4 K/uL	4.8-10.8 K/uL
Neutrophils	89.10%	44-80%
Lymphocytes	7.20%	13-43%
Monocytes	3.20%	2-15%
Eosinophils	0.10%	0-3%
Basophils	0.40%	0-3%
Hemoglobin	7.3 g/dL	12-16 g/dL
Hematocrit	23.20%	37-47%
MCV	70 fL	981-99 fL
Platelet Count	697 K/uL	130-400 K/uL
C-reactive protein	21.3 mg/dL	0.7-1 mg/dL
Iron	22 ug/dL	37-170 ug/dL

**Figure 1:**
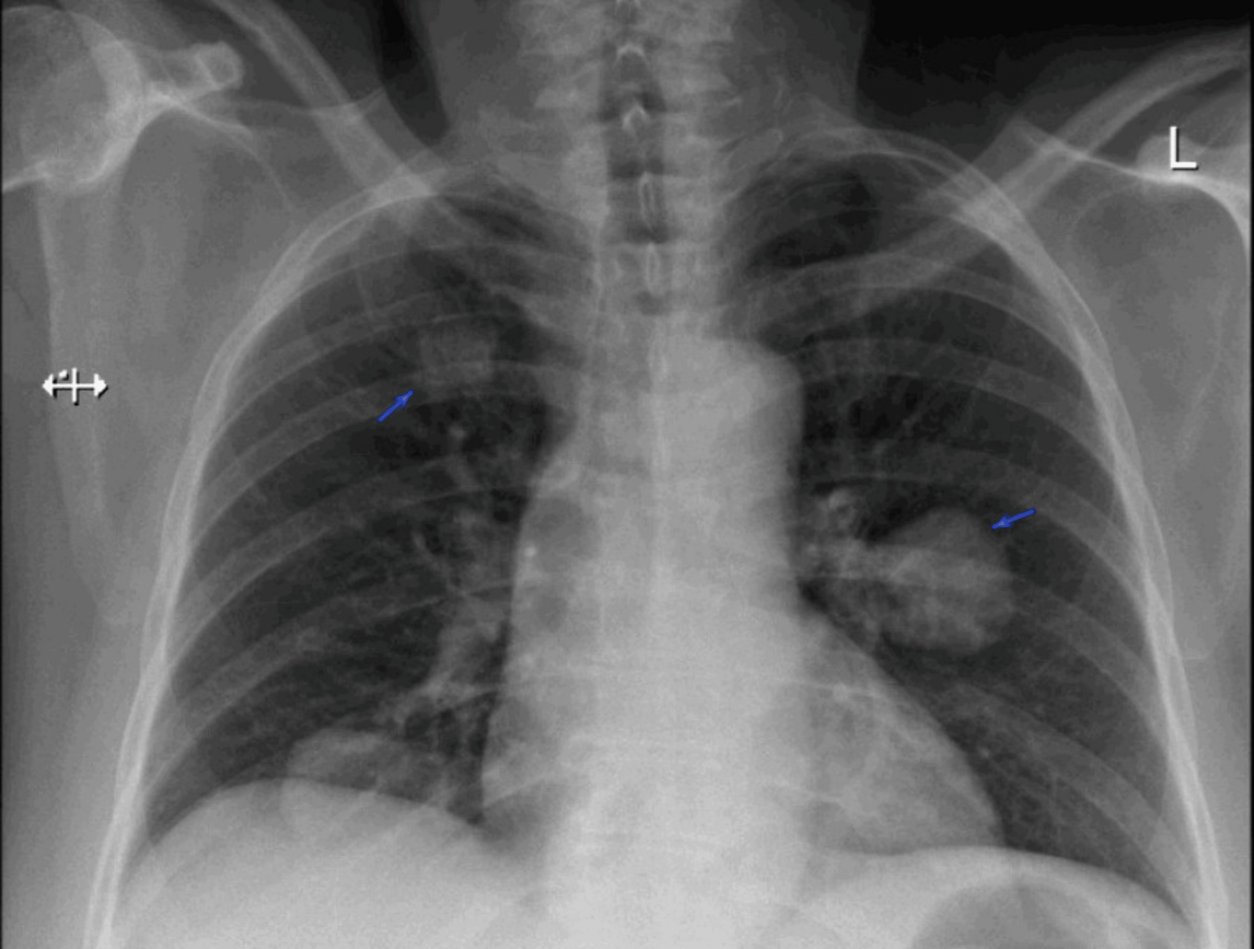
Chest radiograph (PA film) showing bilateral rounded opacities that raised suspicion for metastatic lesions A chest radiograph of the patient (PA film) with bilateral rounded opacities (arrows) PA: posteroanterior

A chest radiograph showed bilateral rounded opacities in the right suprahilar region, the right base, and left perihilar region, which raised suspicion for pulmonary metastasis (Figure 1). Given this finding, further imaging with CT was done. 

**Figure 2:**
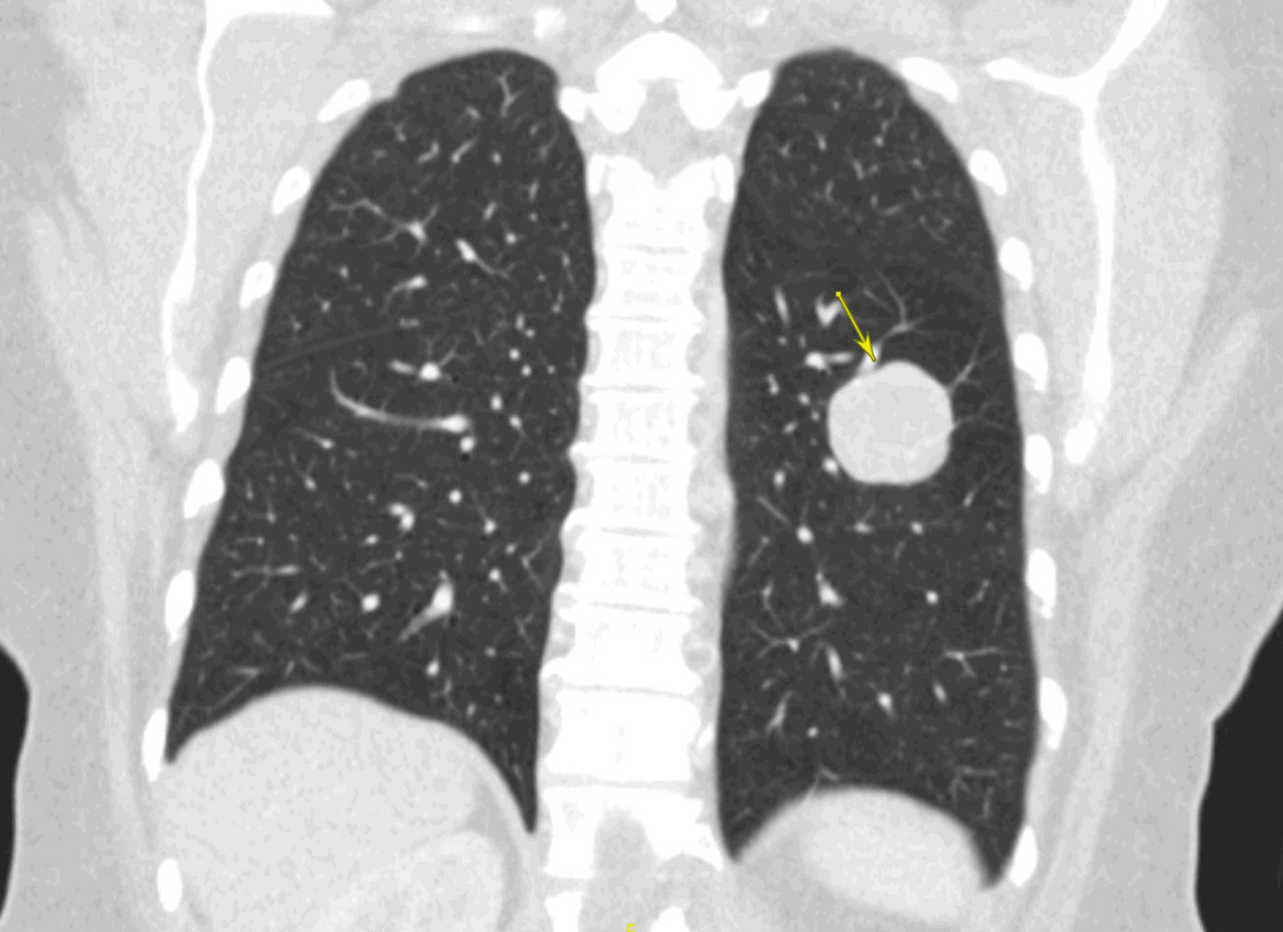
CT scan of the chest showing coronal view of a pulmonary lesion CT Scan of the chest showing a coronal image using lung window showing the largest pulmonary mass in the right middle lobe, 3.8 x 3.3 cm in size (yellow arrow) CT: computed tomography

**Figure 3:**
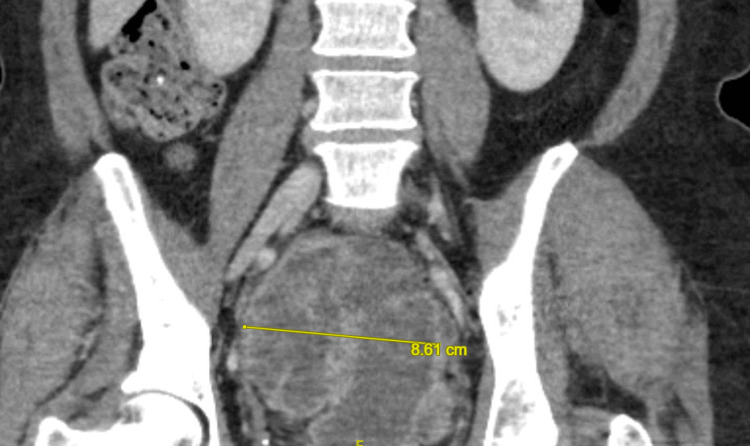
CT scan of the abdomen and pelvis showing large presacral retrorectal mass Sagittal section of a CT scan of the abdomen and pelvis showing a large presacral rectrorectal mass measuring 12.4 x 8.6 x 7.9 cm CT: computed tomography

**Figure 4:**
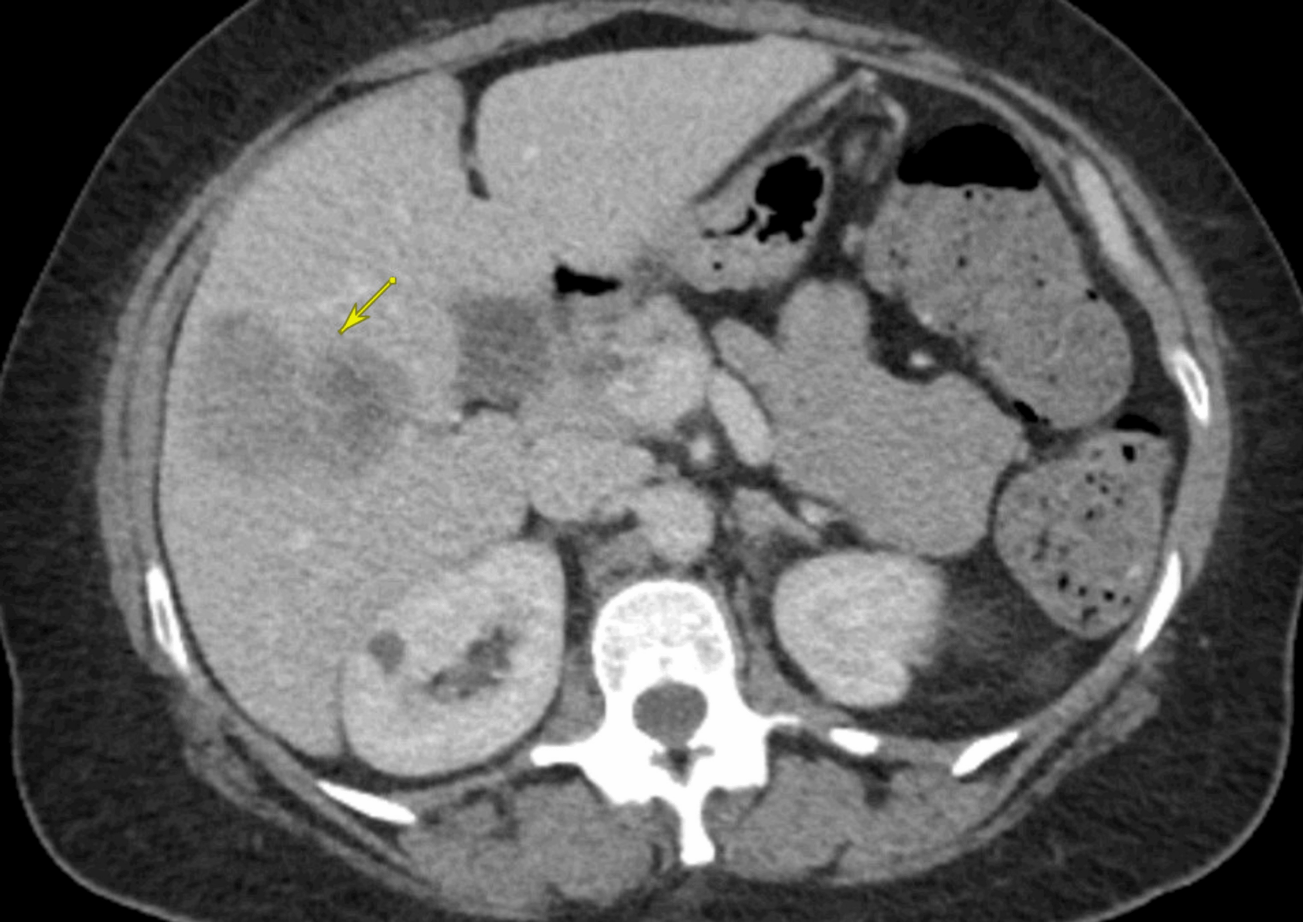
CT scan of the abdomen and pelvis showing hepatic lesion The largest hepatic mass seen on a transverse section of the CT scan of the abdomen and pelvis (yellow arrow) CT: computed tomography

Given the suspicion for malignancy based on the chest radiograph, CT scans of the chest, abdomen, and pelvis with intravenous (IV) contrast were performed. CT of the chest showed masses in the right upper lobe (1.8 x 1.3 cm), right middle lobe (3.8 x 3.3 cm), right lower lobe (2.3 x 1.6 cm), and left lower lobe (3.6 x 2.9 cm), consistent with pulmonary metastases (Figure 2). CT of the abdomen and pelvis revealed a large (12.4 x 8.6 x 7.9 cm) presacral retrorectal mass that was predominantly cystic with extensive septations and mural nodularity (Figure 3). Three heterogeneously enhancing masses with relatively reduced attenuation and enhancement were identified in the right lobe of the liver, the largest measuring 6 x 4 cm, consistent with hepatic metastases (Figure 4). Differentials at the time, based on radiographic imaging, were sarcoma and atypical ovarian cancer. MRI of the brain was negative for metastatic disease.

**Figure 5:**
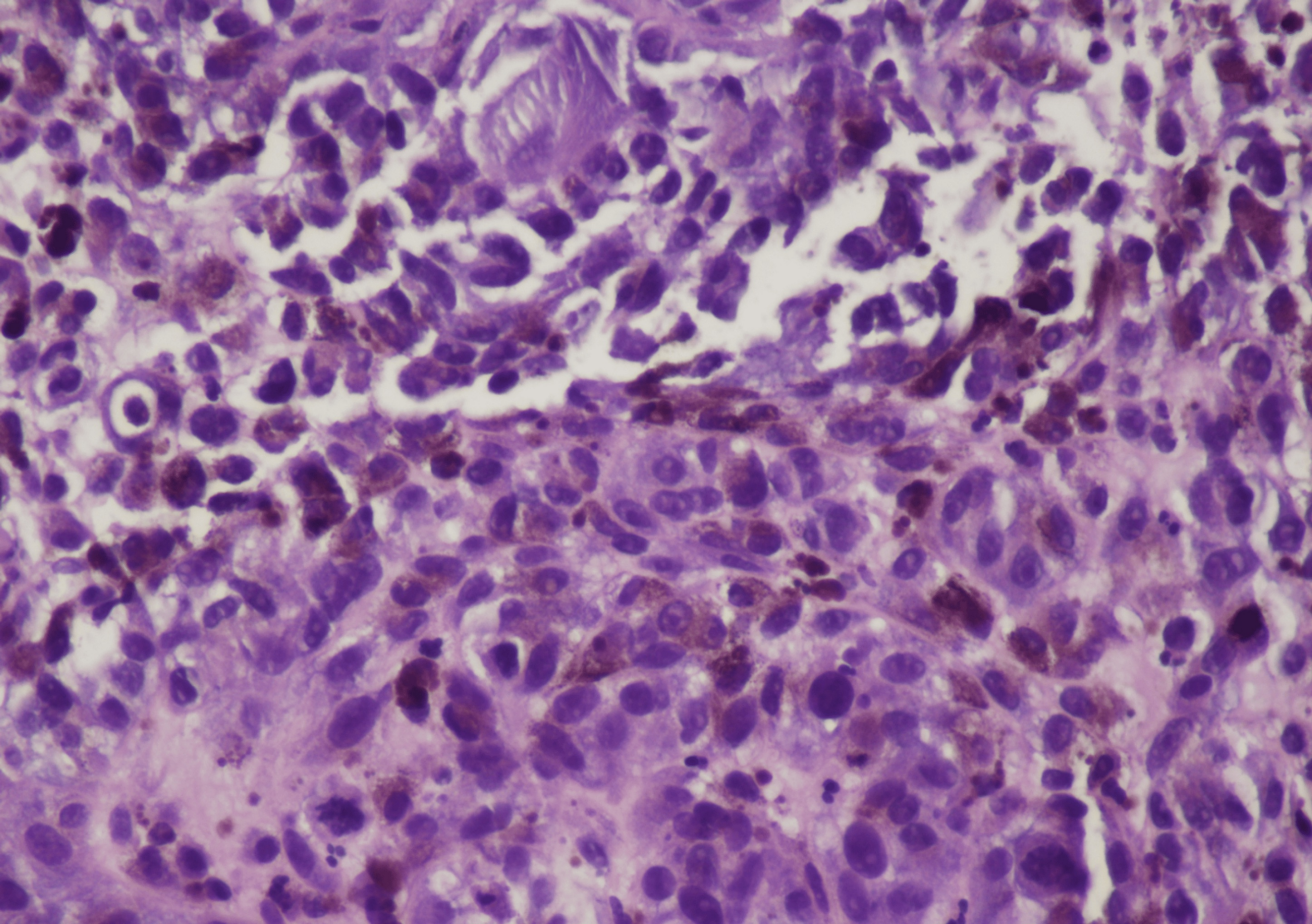
H&E stain of mass biopsied during sigmoidoscopy Numerous melanocytes filled with melanin (black pigment) are seen

**Figure 6:**
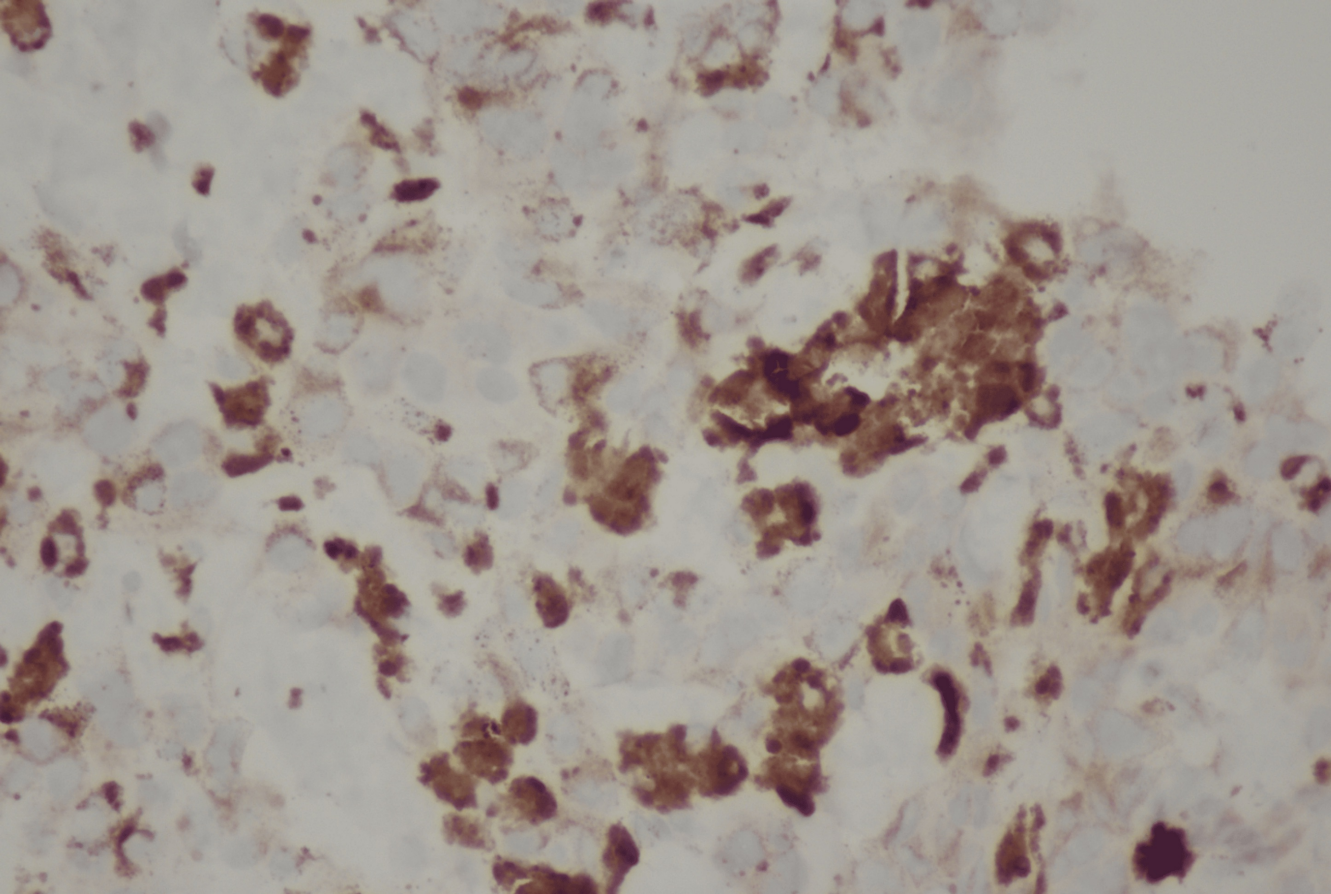
S-100 stain of rectal mass biopsied showing strongly staining cells suggestive of melanoma

The patient was admitted for further workup. Image-guided biopsy of the liver lesion was performed. The biopsy results were as follows: "H&E section shows sheets of tumor cells with enlarged hyperchromatic nuclei. There are diffuse melanin pigments. Immunohistochemical stains were performed with adequate controls. The tumor cells are positive for S100, SOX10, HMB45, MELAN-A (Focal), while negative for AE1/3, SMA, DESMIN, ARGINASE1, and HEPATOCYTE. These findings support the diagnosis of melanoma" (Figures 5-6). Sigmoidoscopy was performed, which revealed an ulcerated mass invading the rectum. Specimens were taken for histopathology, which revealed the following findings: "The H&E section shows sheets of tumor cells with enlarged, eccentric hyperchromatic nuclei with diffuse melanin pigment deposits. Morphological features are consistent with malignant melanoma." 

The patient was found to be a candidate for immunotherapy with ipilimumab and nivolumab and was discharged for outpatient follow-up with Hematology-Oncology. She was not found to be a surgical candidate given the widespread nature of the disease.

However, after two cycles of therapy with ipilimumab and nivolumab on an outpatient basis, her condition began to deteriorate despite treatment, with decreased appetite and eventual near-total cessation of oral intake. She was readmitted to the hospital due to these symptoms. She also experienced severe abdominal pain and became bedbound. Given her poor prognosis, her family elected for comfort measures only at this point. She passed away approximately three months after her initial presentation to the ED, with bowel obstruction secondary to the rectal melanoma as the likely underlying cause of death.

## Discussion

Melanomas presenting as pelvic masses are rare. The most common sites of melanoma are the skin, the eyes, and the mucosal surfaces of the body [3,4,5]. These commonly observed locations underscore the unusual nature of a primary melanoma arising in the retrorectal space. While anorectal melanomas, a subset of mucosal melanomas, have been reported to cause rectal bleeding, our case is particularly notable for a primary retrorectal melanoma presenting with this symptom. This distinction in anatomical origin may influence the clinical presentation and diagnostic pathway.

The initial diagnosis in our patient was delayed, likely due to the absence of laboratory investigations during the first ED visit and the retrorectal mass not being appreciated on initial digital rectal examination (DRE). This highlights a potential pitfall in the evaluation of rectal bleeding, where more common benign etiologies like hemorrhoids often take precedence. The difficulty in palpating retrorectal masses on DRE, especially when they are smaller or located higher in the pelvis, can contribute to diagnostic delays [7]. Given that the age for colon cancer screening in regular patients is considered to be 45, and as our patient was beyond that age range, consideration should have been taken at the time in the ER, as a simple complete blood count would have prevented the diagnosis from being missed. A high index of suspicion for less common causes is crucial, particularly in cases with atypical presentations or persistent symptoms.

The mass, as seen on radiological imaging, was described as predominantly cystic with extensive septation and mural nodularity. CT is not a good imaging modality to differentiate between malignant melanoma and other possible differentials. Ovarian malignancies, a possible differential are better evaluated with ultrasonography as it can also examine tumor vascularity [8]. Gastrointestinal stromal tumors (GISTs), another differential, are usually seen as lobulated and heterogeneous tumors [9]. Soft tissue sarcomas, yet another differential, have extremely varied appearances on imaging, ranging from being homogenous to larger ones having large areas of necrosis [10]. In our patient's case, MRI could have possibly helped in seeing if a differential diagnosis like a GIST was more likely, and ultrasonography could have helped evaluate for possible ovarian carcinoma; however, ultimately, all of the above tumors can have varied appearances, and only a biopsy would have confirmed the diagnosis. Hence, further imaging was not pursued at the time, and the decision was made to proceed directly with the biopsy. 

The diagnosis of melanoma in our patient was strongly supported by the S-100 stain, along with other melanocytic markers such as Melan-A and HMB-45 identified in the biopsy. S-100 is a widely recognized marker for melanoma, although not entirely specific [11]. The co-expression of Melan-A and HMB-45 further strengthens the diagnosis, as these markers are more specific to melanocytic differentiation [11,12].

Treatment options for metastatic melanoma have evolved significantly. Immunotherapies, including combination regimens such as a PD-1 inhibitor (nivolumab or pembrolizumab) with a CTLA-4 inhibitor (ipilimumab) or a LAG-3 inhibitor (relatlimab), and single agents like pembrolizumab or nivolumab, have demonstrated improved survival outcomes in many patients. Targeted therapies, such as imatinib for patients with c-KIT mutations, and BRAF inhibitors (e.g., dabrafenib, vemurafenib, encorafenib), often combined with MEK inhibitors (e.g., trametinib, binimetinib) for BRAF-positive tumors, offer effective treatment for specific molecular subtypes. Combination chemotherapy regimens, such as CVD (cisplatin, vinblastine or vindesine, and dacarbazine) and Dartmouth (carmustine, dacarbazine, cisplatin, and tamoxifen), are also used, though often with lower response rates and less durable responses compared to immunotherapy and targeted therapy in appropriate subsets [6,13,14]. BRAF mutation testing was performed for this patient, guiding potential treatment strategies if a mutation had been identified. Since our patient was not a surgical candidate due to the widespread nature of her disease, she was started on immunotherapy. Chemotherapy was not pursued, as, although her tumor had progressed on immunotherapy, her performance status by that point was such that it was prohibitive to start chemotherapy. 

The prognosis for metastatic melanoma remains generally poor, with a median survival of approximately 20 months [6,14,15]. However, it is important to note that this figure represents an average, and outcomes can vary significantly based on factors such as the extent of the disease, performance status, and response to treatment, particularly with the advent of newer therapies [15]. The rapid deterioration observed in our patient, despite initial immunotherapy, underscores the aggressive nature of some metastatic melanomas and the challenges in achieving durable responses in all individuals.

## Conclusions

Retrorectal melanoma presenting with rectal bleeding is uncommon and can be initially misdiagnosed as more benign conditions, such as hemorrhoids, as occurred in this case. Therefore, in cases of persistent or unusual rectal bleeding, especially when initial examinations are inconclusive, a thorough workup including imaging and laboratory investigations is crucial to avoid delayed diagnosis of rare but serious conditions like retrorectal melanoma.
